# Long-term potentiation: 50 years on: past, present and future

**DOI:** 10.1098/rstb.2023.0218

**Published:** 2024-07-29

**Authors:** W. C. Abraham, T. V. P. Bliss, G. L. Collingridge, R. G. M. Morris

**Affiliations:** ^1^ Department of Psychology, Brain Health Research Centre, University of Otago, Dunedin 9054, New Zealand; ^2^ Group leader emeritus, The Francis Crick Institute, London NW1 1AT, UK; ^3^ Lunenfeld-Tanenbaum Research Institute, Mount Sinai Hospital, Toronto, Ontario M5G 1X5, Canada; ^4^ Tanz Centre for Research in Neurodegenerative Diseases, Department of Physiology, University of Toronto, Toronto, Ontario M5T 0S8, Canada; ^5^ Centre for Discovery Brain Sciences, Edinburgh Neuroscience, School of Biomedical Science, University of Edinburgh, Edinburgh EH8 9JZ, UK

**Keywords:** long-term potentiation, long-term depression, synaptic plasticity, learning, memory, cognitive disorders, LTP

## Abstract

We introduce and summarize reviews and research papers by speakers at a discussion meeting on ‘Long-term potentiation: 50 years on’ held at the Royal Society, London, on 20–21 November 2023. The meeting followed earlier discussion meetings marking the 30th and 40th anniversaries of the discovery of long-term potentiation. These new contributions give an overview of current research and controversies in a vibrant branch of neuroscience with important implications for our understanding of the neurobiological basis of many forms of learning and memory and a wide spectrum of neurological and cognitive disorders.

This article is part of a discussion meeting issue ‘Long-term potentiation: 50 years on'.

## 1. Introduction

Long-term potentiation (LTP), the activity-dependent long-lasting enhancement of synaptic efficacy, has fascinated neuroscientists for over 50 years now, and not surprisingly, it has had an immense impact on neuroscience generally. This interest was sparked by back-to-back publications in the *Journal of Physiology* by Bliss and Lømo [1] and Bliss and Gardner-Medwin [2] describing the phenomenon in the anaesthetized and awake rabbit, respectively. These reports built on the initial observations of Terje Lømo working in Per Andersen’s lab and reported in an abstract form in 1966 [3]. The question of its function was raised at the time—‘Whether or not the intact animal makes use in real life of a property which has been revealed by synchronous, repetitive volleys to a population of fibres the normal rate and pattern of activity along which are unknown, is another matter’ [1]. Equally cautiously—‘Since the phenomenon is present in healthy unanaesthetized animals, it is at least possible that its mechanism could underly some form of plasticity under normal conditions in the hippocampus’ [2]. The ease with which LTP could be induced and these conjectures about its significance for memory launched a subfield of neuroscience that has continually grown over the ensuing decades. Fifty years later, in November 2023, a number of prominent researchers probing the mysteries of LTP gathered at the Royal Society in London to present their latest findings and discuss current issues surrounding the field. The collection of papers in this special issue reflects those topical discoveries and discussions.

## 2. LTP50 in context

Before providing an overview of the meeting and the papers presented there, it may be helpful to place current studies in the context of developments over the past 50 years. It is instructive to see which issues attracted early interest, which of these the field has moved on from, which others continue to bedevil us and which new lines of work have branched into today’s world. This light-touch review, done roughly by decade, serves to provide context for the coming reports from the meeting. There are many nuances, strands and controversies that we do not have space to cover but a range of in-depth reviews abound for those interested.

The publications in 1973 were quickly seized upon by a small number of groups, and the rest of the 1970s was largely taken up with research aiming to replicate and extend those initial findings. Finding stimulation protocols that would optimize the generation of LTP to enable reliable mechanistic research to be undertaken was a high priority, along with testing predictions of the Hebb postulate^1^ such as synapse specificity, associativity and the requirement or not for postsynaptic action potentials. The first studies of morphological changes at synapses were also launched, while the development of the hippocampal slice preparation for electrophysiological and biochemical studies signalled a shift towards a much more controlled, if less physiological, experimental setting for addressing LTP mechanisms [4].

The 1980s saw the beginnings of a debate about LTP mechanisms that continues today in one form or another. Namely, is the final expression mechanism of LTP presynaptic (e.g. a sustained increase in transmitter release), postsynaptic (e.g. an increase in the number or efficacy of glutamate receptors) or does it involve both sides of the synapse (e.g. having more synapses)? Evidence for increased transmitter release after induction of LTP was contrasted with requirements for postsynaptic depolarization for LTP to be induced in the first place. Mechanistic studies, largely using pharmacological tools, pointed to a change in the population of postsynaptic AMPA receptors. A major breakthrough for LTP research was the discovery that postsynaptic *N*-methyl-d-aspartate (NMDA) glutamate receptors are vital for the induction of LTP yet without contributing to basal synaptic transmission. Interestingly, NMDA receptors were shown not to contribute to LTP expression or maintenance when inhibited post-induction, whereas de novo protein synthesis was found to be important for its maintenance but not induction. With the gradual development of ever more selective glutamatergic ligands, the field had access to drugs for selectively manipulating LTP at different stages. Their development also aided the launch of causal studies into the behavioural relevance of LTP whereby the selective block of different stages of LTP could be investigated in relation to corresponding changes in memory function. Long-term depression (LTD) also began to attract interest, although this form of plasticity was thought to be largely heterosynaptic in nature at this juncture.

The next decade saw a rapid rise in LTP experimentation addressing in much more detail its induction and expression mechanisms. The range of induction protocols was significantly expanded when it was shown that low-frequency pairing of synaptic input activity with postsynaptic cell firing could induce either LTP or LTD depending on the precise timing of the pre- and postsynaptic events, a phenomenon known as spike-timing-dependent plasticity (STDP). Roles for kinases such as calcium/calmodulin-dependent protein kinase II, protein kinase A and protein kinase C in the induction and early expression of LTP were identified. The presynaptic–postsynaptic debate got serious, with intensive studies of the molecular mechanisms of postsynaptic glutamate receptor trafficking that were complemented by the revelation of the existence of ‘silent synapses’ that could be ‘woken up’ by adding α-amino-3-hydroxy-5-methyl-4-isoxazolepropionic acid (AMPA) receptors. Meanwhile, further evidence for altered transmitter release probability was obtained, driving the search for candidate retrograde messengers from postsynaptic to presynaptic sites, such as nitric oxide and arachidonic acid. The role of protein synthesis in maintaining LTP over hours or more became more interesting when it was revealed that proteins could be shared across dendritic sites through synaptic tag and capture (STC) mechanisms. Also at this time, the first evidence emerged that an atypical protein kinase C, PKMζ, might play a critical ongoing role in LTP maintenance. Notably, an antagonist of PKMζ, termed ZIP, provided the first pharmacological agent that could abolish LTP at any time after its induction.

Another layer of molecular control was revealed when it was shown that gene transcription contributed to longer-lasting forms of LTP, mediated by expression of immediate early genes and generation of their inducible transcription factors that regulate other late-response genes. Alongside such work, other related plasticity phenomena began to come under more scrutiny following the development of protocols for inducing homosynaptic LTD, the discovery of metabotropic glutamate receptor (mGluR)–LTD and the realization that the ability of any given protocol to generate LTP or LTD was strongly influenced by the history of prior activity at those synapses, mediated by a family of mechanisms termed metaplasticity. These findings, together with new tools such as two-photon uncaging of molecules at synapses, calcium imaging combined with two-photon microscopy that allowed transmission at single synapses to be monitored before and after the induction of LTP, gene targeting methods and transgenic mice, collectively provided rich resources underpinning the increasingly important aim of understanding how LTP and LTD are mediated and how they contribute to information processing and memory.

Across these first three decades of research, there was an exponential increase in the breadth and depth of synaptic plasticity research. It seemed that at last an understanding of the neural mechanisms of learning and memory—the driver for this interest—might be within our grasp. This sparked in 2003 the first LTP celebration at the Royal Society, the LTP30 Discussion Meeting hosted by Bliss, Collingridge and Morris. Looking at the papers in the associated Special Issue (https://royalsocietypublishing.org/toc/rstb1990/2003/358/1432), one can see how far the field had come since the early days, how much was still being discovered and the outstanding issues to be addressed. The evidence for expression mechanisms on both sides of the synapse continued to grow, studies of the plasticity of synaptic structure gained maturity, and how long LTP could last (at least up to a year in rats) and by what mechanisms was better understood. It was also evident that there was now a substantial body of evidence that strongly supported the synaptic plasticity and memory (SPM) hypothesis. On top of these basic research studies, new lines of research were taking off. Specifically, interest was gathering with respect to the translational relevance of disorders of synaptic plasticity—for example, how LTP was impaired in models of neurological diseases such as Alzheimer’s disease (AD) and by disease-associated molecules such as amyloid-β.

Ten years later in 2013 came the LTP40 celebration. The Special Issue from this meeting published in 2014 (https://royalsocietypublishing.org/toc/rstb/2014/369/1633) was again a mixture of new light on old questions along with a discussion of newly uncovered aspects of synaptic plasticity. ‘Pre-ists’ and ‘Post-ivists’ were still at it, although there seemed to be a growing agreement that both presynaptic and postsynaptic expression mechanisms could be in play, depending on the LTP induction paradigms. Meanwhile, other features were attracting interest such as the special role of GluA2-lacking AMPARs in the early maintenance of LTP and the multiplicity of overlapping short-term and long-term potentiation mechanisms that could be dissected. For example, LTP can occur at excitatory synapses on inhibitory interneurons as well as at inhibitory synapses on excitatory principal cells, while glial cells play a regulatory role in plasticity and metaplasticity. A key development was looking beyond the hippocampus to other brain areas. LTP and LTD phenomena were being studied throughout cortical and subcortical brain areas, amplifying a trend already occurring in the previous decade, with findings in the amygdala and cortex being prominent. Optogenetics was a major addition to the toolkit, and its use helped underpin the growing support for the SPM hypothesis. Perhaps not surprisingly, there was a significant uptick in the study of plasticity—or its impairment—in disease conditions, with studies in the context of AD, autism, intellectual disability, chronic pain and autoimmune encephalitis being represented at the meeting.

Now, 50 years on, the study of LTP and associated plasticity phenomena remains a cornerstone of contemporary neuroscience research. There was no lack of interest in or material for another decade celebration of the field, again held at the Royal Society. Here, we briefly touch on the many papers in this Special Issue that arose from that 2-day meeting, where it will become apparent that the themes of research from the previous 50 years continue to expand, hook up and diverge in ever more interesting ways. Here, we have divided the papers in this Special Issue into three broad categories: plasticity mechanisms, behavioural relevance of synaptic plasticity and plasticity-related disease mechanisms.

## 3. Plasticity mechanisms

LTP or LTP-like phenomena are ubiquitously observed across the animal kingdom, but rodent LTP is far more intensively studied and understood than in other species. Given that non-human primates are difficult to source and study for LTP mechanisms, Song *et al.* [5] investigated cingulate cortex LTP in a close family member of primates, the tree shrew. A comparison between tree shrews and C57BL/6 mice revealed a strong similarity of effects and mechanisms at glutamatergic synapses, although interestingly the tree shrews exhibited greater LTP responses to theta-burst stimulation (TBS) patterns than the mice.

Regarding its induction, it is well established that LTP at many synapses involves the synaptic activation of NMDARs. Li *et al.* [6] added a new twist demonstrating the importance of NMDAR splicing. Through genetic manipulations, they separately expressed NMDA receptors built around either GluN1a (lacking the exon 5-encoded N1 cassette) or GluN1b (exon 5 expressing). They found that GluN1a–NMDA receptors trigger greater LTP and are subject to regulation by Src tyrosine kinase, while GluN1b LTP is Src-resistant. Thus, alternative splicing of GluN1 leads to substantial differences in LTP and its regulation by Src.

The expression mechanisms of LTP continue to be extensively studied. On the postsynaptic AMPA receptor side of the equation, an increased complement of receptors has long been shown to accompany LTP induction. Nowacka *et al.* [7] review the increasingly complex literature regarding mechanisms by which receptors are provided to the active zones in an activity-dependent way and remain there to underpin at least one key aspect of LTP expression. Single-particle tracking by the Choquet laboratory has revealed a surprising diffusibility of AMPA receptors, but also trapping, in the postsynaptic density (PSD) to mediate LTP. Importantly, early LTP can be mediated by the targeting of GluA2-lacking receptors to the synapse. Because these receptors are permeable to calcium ions and support protein synthesis-dependent LTP, Koek *et al.* [8] questioned whether calcium-induced calcium release (CICR) underpinned STC mechanisms. Using inhibitors of both inositol trisphosphate receptors and ryanodine receptors on the endoplasmic reticulum, they were able to show that indeed CICR is of fundamental importance for these heterosynaptic interactions. Morphologically, spine head sizes scale with the AMPA receptor complement. Harris *et al.* [9] review the structural literature that provides evidence supporting a model in which LTP induction is correlated with a conversion of nascent zones (with a PSD but no opposing vesicles), followed by an expansion of the PSD by development of new nascent zones that can support further LTP at those synapses in the future. In contrast, LTD appears to involve the loss of weakly formed active zones, accompanied by spine shrinkage.

There have been many studies of presynaptic contributions to LTP expression, and Yamamoto *et al.* [10] add to this literature by studying presynaptic LTP in the insular cortex. This form of corticocortical LTP requires activation of kainic acid receptors and release of nitric oxide, presumably acting as a retrograde messenger, as demonstrated by patching of two connected insular cortex neurons. Synaptic transmission is mediated by an action potential-mediated increase in free intracellular calcium presynaptically, triggering vesicle release. Schmidt *et al.* [11] employed presynaptic calcium imaging of CA3 boutons to reveal the surprising finding that presynaptic NMDA receptors play a regulatory role in the calcium response depending on the subunit composition. GluN2A-containing receptors enhance the calcium response, while GluN2B-containing receptors do the opposite, all via regulation of SK calcium-dependent potassium channels. Such effects likely modulate the presynaptic transmitter release component of LTP expression. How do the presynaptic and postsynaptic expression mechanisms of LTP individually affect cell outputs and network activity? This question was addressed by Savtchenko and Rusakov [12] using computational modelling of a CA1 pyramidal cell in isolation and in a circuit of cells. Calculating proportionally equal increases in the presynaptic release probability and the postsynaptic response revealed a far larger effect on single cell firing by the postsynaptic manipulation, while the increase in network firing synchronization was greater following an increase in release probability.

Short-term potentiation (STP) is a part of the LTP family that is induced rapidly in response to high-frequency stimulation and decays in an activity-dependent way. The use-dependent decay of STP is found in both dorsal and ventral hippocampus (Ingram *et al*. [13]) and endows synaptic plasticity mechanisms with a hitherto under-appreciated mechanism. How the storage of potentiation is regulated when no stimulation occurs is unknown. Ingram and Volianskis [14] report experiments demonstrating that activation of metabotropic glutamate receptors (mGluRs) plays critical roles in the maintenance of the STP across time when there is no synaptic activity. In other work involving mGluRs, Mockett *et al.* [15] show that group I mGluRs stimulate protein synthesis via activation of transmembrane protein sheddases such as ADAM17. Blocking these sheddases prevents the protein synthesis-dependent effects of group I mGluR-mediated metaplastic priming of LTP persistence and induction of at least some forms of mGluR–LTD.

## 4. Behavioural relevance of synaptic plasticity

Understanding the possible roles of the various forms of activity-dependent synaptic plasticity in learning and memory has been a goal since the discovery of LTP. But first, in order for synaptic plasticity to be relevant to learning and memory, it needs to be evident in response to experience and to correlate with behavioural or cognitive changes. Regele-Blasco and Palmer [16] review the literature on *in vivo* plasticity of synapses, dendrites and cell firing that is observed for pyramidal neurons found across many brain regions. That circuits are capable of plasticity is indisputable, but the role of plasticity for behaviour, even for the same type of plasticity, is likely to depend on the brain region and circuit in which it is produced. More generally, synaptic plasticity mediates different functions when embedded in different local circuits. There is, for example, growing evidence that it helps mediate pattern separation in the dentate gyrus, sequence learning in CA3 and event–context associations in CA1.

Gall *et al.* [17] review evidence supporting this view that circuits matter even within the confines of the hippocampus. For example, LTP at CA3–CA1 synapses appears to work in support of unsupervised learning of episodic information, whereas LTP at lateral perforant path synapses in the dentate gyrus may mediate reward-based learning (such as during operant conditioning). Importantly, they also note that LTP at CA3–CA1 synapses differs between males and females in terms of oestrogen dependence, amplitude and developmental stage, indicating that the role of LTP in learning is nuanced and difficult to capture across all settings. Adding to these complexities, Manahan-Vaughan and Hegena [18] review rodent *in vivo* studies of both LTP and LTD and discuss how these plasticity phenomena are affected by behavioural manipulations. They conclude that LTP appears to enable the generation of a record of spatial experience that may serve as an associative schema that can be re-used to expedite or facilitate subsequent learning. In contrast, LTD may enable modification and dynamic updating of this representation, rendering it distinguishable from other similar representations. With respect to episodic information processing, Sekeres *et al.* [19] consider whether and how memory schema are updated by new information (again presumably by plasticity processes). They propose a continuum from very familiar to very novel new information that determines the extent to which the hippocampus becomes involved in the updating of existing schemas versus the creation of a new one through its communication with the prefrontal cortex.

There are other lines of evidence pointing to the role of LTP in memory being brain region-dependent. For example, plasticity in the motor cortex could well be related to motor learning. Kim *et al.* [20] illustrate this point beautifully using genetic labelling to identify activated cells in M2 and M1 regions and dual eGRASP labelling to identify synaptic connections from M2 to M1. They found that rotarod learning was accompanied by increases in synaptic density in the M2–M1 connections between cells activated during learning, but only in mice that learnt successfully. Another contribution took a more indirect approach to the general question of the neural mechanisms of learning and memory but in which synaptic plasticity could still be inferred to play a role. Park *et al.* [21] used auditory threat conditioning that led to two concomitant behavioural responses to subsequent presentation of the auditory stimulus, freezing and ultrasonic vocalizations (USVs). During retrieval, optogenetic activation or inactivation of lateral amygdala engram cells active during training elicited effects on the freezing response and the power spectral density of the USVs that were comparable to those elicited by the natural stimulus.

Plasticity in connections between brain regions must also be considered with respect to Hebbian cell assemblies or engram circuits in contemporary parlance. In context fear conditioning, for example, plasticity centred on the dentate gyrus mediates learning about the context but the synaptic plasticity-mediated association of this learnt representation with fear occurs in the basolateral amygdala. Similarly, Fayed *et al.* [22] extend the role of LTP to encompass not just learning at the time of an experience but also the plasticity connections between neural circuits that occur while the brain is ‘idling’, for example, during sleep or quiet wakefulness. The generation of LTP between ensembles of co-active engram cells during ‘idling’ (that might not normally be co-active under more aroused states) could be a basis for creative thinking. Another way of binding different memories in a circuit is through the STC mechanisms of protein synthesis-dependent LTP, which are thoroughly reviewed by Bin Ibrahim *et al.* [23]. In particular, this mechanism may be underpinning the strengthening of what would normally become a weak memory when temporally coincident with a stronger or emotionally charged experience. Impairment in STC mechanisms could also contribute to cognitive impairments across a range of disorders including stress, sleep deprivation, ageing and AD.

## 5. Plasticity-related disease mechanisms

As noted above, AD has attracted much attention from plasticity researchers given the early involvement of memory impairments. There are likely many contributing causes to AD, including glial contributions, but impairments in LTP and LTD are prominent among them. How the oligomeric species of amyloid-β and tau might generate such impairments mechanistically is still not resolved, especially for tau. Addressing this question, Ondrejcak *et al.* [24] found that both oligomerized recombinant tau and tau extracts from post-mortem AD and Pick’s disease brains cause a rapid and persistent inhibition of LTP in intact rat hippocampus, an effect exacerbated by amyloid precursor protein over-expression. That tau monoclonal antibodies could reverse the LTP inhibition even when given two weeks after tau injections speaks to the ongoing nature of the effects on LTP by oligomeric tau. LTP-like impairments in Alzheimer’s patients have also been observed. Naveed *et al.* [25] used the transcranial magnetic stimulation protocol of paired associative stimulation (PAS) in the dorsolateral prefrontal cortex of human subjects and found an impairment in the PAS-generated LTP that correlated with the thickness of that cortical region. On the other hand, superior longitudinal fasciculus mean diffusivity in AD patients correlated with PAS–LTP impairment when repetitive PAS was delivered over a 4-week period.

Fragile X syndrome (FXS) is also characterized by impaired LTP and LTD, depending on the brain region studied. FXS is also associated with reduced circulating levels of the neurohormone adiponectin. Thacker *et al.* [26] found that a brief administration of adiponectin to dentate gyrus slices completely rescued LTP and LTD in the *fmr1*-KO model of FXS, alongside an increase in the expression and phosphorylation of GluA1. This study thus revealed a previously unknown important role for this hormone in the disease. LTP and STP are also impaired in area CA1 of these mice, but the extent of the impairment in plasticity, along with an impairment of contextual fear conditioning, appears to be dependent on the specific breeding strategy and housing conditions for the mice, as presented by Volianskis *et al.* [27]. Changes in NMDA receptor subunit expression and the rescue of LTP by an NMDA receptor-positive allosteric modulator were also dependent on this ‘cage effect’.

A disease with a more complex genetic basis is autism spectrum disorder (ASD). Of the many models of ASD, Lee *et al.* [28] used the Shank2 knockout mouse model. Shank proteins play a vital role in PSD organization. Dietary delivery of zinc for 6–8 weeks was found to reverse hyperactivity and impairments in social behaviour, without affecting deficits in working memory or the AMPA/NMDA receptor-mediated synaptic transmission ratios. This work adds to the literature on the potential therapeutic value of zinc supplementation in treating ASD. Clinical depression, on the other hand, may have a closer link to altered NMDA receptor function. Jiang *et al.* [29] review the literature regarding the effectiveness of the non-competitive NMDA receptor antagonist ketamine in treating depression, in both humans and animal models. The available data support a strong therapeutic effect by ketamine, but it remains uncertain whether this effect is achieved by NMDA receptor/channel antagonism or by one of the many other side effects of ketamine, given that other NMDA receptor antagonists have not been as effective. This also raises the question of whether a reduction in LTP, as is produced by ketamine, should be considered a therapeutic target for major depression.

## 6. Looking ahead

As we once again gaze into the crystal ball, we can reminisce on how accurate (or not) our predictions for the past decade have been. We hoped that the pre- versus postsynaptic expression of NMDAR–LTP debate would be finally resolved. It can confidently be concluded that the great majority of neuroscientists now agree that modifications on both sides of the synapse underlie this family of potentiation mechanisms. The interest lies now in understanding the locus of expression of the various components of synaptic plasticity. We predict that this will be fully resolved to the satisfaction of most scientists over the course of the next decade.

We predicted more investigations into the signalling mechanisms of the various forms of synaptic plasticity and metaplasticity. Indeed, much has indeed been learnt since LTP40, some of which is covered in this issue. No doubt this quest will continue, and the core cellular mechanisms of STP1, STP2, LTP1, LTP2, LTP3, de novo LTD, depotentiation, heterosynaptic LTP, homosynaptic metaplasticity, heterosynaptic metaplasticity, etc. will be unravelled. This will indeed represent a significant achievement as we delve even deeper into the realm of the functional significance of LTP.

By the time of LTP40, the SPM hypothesis had been widely accepted. The major achievements in the characterization of hippocampal engrams were noted, as was the need to develop methods to study synaptic engrams. It was stated that ‘However, an experiment in which spatial patterns of synaptic change are activated or re-activated, as distinct from the neurons within which these patterns are expressed, remains unachieved’. The past decade has seen a remarkable progress in the development of methods to study synaptic engrams—most notably the dual eGRASP approach. We foresee a progressive shift from the study of cellular engrams to synaptic engrams.

We noted how it had become established that dysfunction of synaptic plasticity is at the heart of memory dysfunction in many major brain disorders. Not surprisingly, the past decade has seen an explosion of studies aimed at characterizing plasticity deficits in mouse models of human disease. The present issue contains such examples in the context of AD, fragile X, autism and chronic pain. There is little doubt that the study of synaptic plasticity in animal models of disease will intensify. It is also likely that more plasticity studies will be conducted in human volunteers and patients with the aim of improving methods for enhancing LTP-like phenomena in order to drive improvements in cognition and mood.

Many of the advances over the past 10 years have been driven by methodological developments, notably optogenetics. We can predict that the same will be true over the next 10 years. In particular, developments in imaging, such as super-resolution microscopy and cryo-EM, are enabling the synapse and its molecular components to be investigated in hitherto unprecedented detail. Along with other technical advancements no doubt yet to come, these techniques will enable a more detailed description of the molecular basis of synaptic plasticity. At the other end of the spectrum, new technologies for live imaging during behavioural tasks of spine plasticity in widespread circuitries in the brain will help us better understand the role of LTP and LTD in shaping and storing information.

Recently, some of us identified 10 major unresolved questions regarding synaptic plasticity in the hippocampus that we anticipate will be answered over the next decade or two [30]. Using those questions as a template, we adapt them here for this wider purpose. These are:

— How do the various components of synaptic plasticity interact with intrinsic plasticity to preserve different kinds of memory in different brain regions?
— How important is metaplasticity (in particular homosynaptic priming and STC) for memory formation?
— What is the role of synaptogenesis in memory storage?
— How does the plasticity of inhibitory neurons impact mnemonic function?
— How do homeostatic plasticity and synaptic plasticity interact during memory formation?
— How do neuromodulatory and neurohormonal factors, individually and in combination, regulate synaptic plasticity to impact learning and memory?
— How do glial cells contribute to and regulate plasticity phenomena?
— What is the contribution of altered synaptic plasticity to neurodevelopmental disorders?
— What is the contribution of altered synaptic plasticity to neuropsychiatric disorders?
— What is the contribution of altered synaptic plasticity to acute and chronic neurodegenerative conditions?

There are, of course, other important questions related to synaptic plasticity that will keep researchers busy over the next decade.

Indeed, another major development that is impacting all fields of science is artificial intelligence (AI). How AI will specifically influence synaptic plasticity research is hard to predict. But it’s interesting to note that, in essence, LTP is to natural intelligence (NI) what the transistor is to AI. Transistors drive the chips that underpin AI. LTP drives the synaptic plasticity that underlies NI. Our cognitive processes have enabled us to generate AI, and our knowledge of the biological processes underlying memory formation has deeply informed machine learning and AI algorithms. Perhaps AI will return the favour by providing the definitive description of LTP, its mechanisms and its role in memory formation in both health and disease.

## Data Availability

This article has no additional data.

## Footnotes

^1^
When an axon of cell A is near enough to excite a cell B, and repeatedly and persistently takes part in firing it, some growth process or metabolic changes takes place in one or both cells such that A’s efficiency in firing B is increased.’ [4]
